# Tissue Biomarkers for Prostate Cancer Radiation Therapy

**DOI:** 10.2174/156652412800792589

**Published:** 2012-07

**Authors:** PT Tran, RK Hales, J Zeng, K Aziz, T Salih, RP Gajula, S Chettiar, N Gandhi, AT Wild, R Kumar, JM Herman, DY Song, TL DeWeese

**Affiliations:** 1 Department of Radiation Oncology and Molecular Radiation Sciences, Sidney Kimmel Comprehensive Cancer Center, Johns Hopkins Medicine, Baltimore, MD, USA; 2 Department of Oncology, Sidney Kimmel Comprehensive Cancer Center, Johns Hopkins Medicine, Baltimore, MD, USA; 3 Department of Urology; Sidney Kimmel Comprehensive Cancer Center, Johns Hopkins Medicine, Baltimore, MD, USA

**Keywords:** Biomarkers, prostate cancer, radiation therapy, RTOG, salvage radiation therapy.

## Abstract

Prostate cancer is the most common cancer and second leading cause of cancer deaths among men in the United States. Most men have localized disease diagnosed following an elevated serum prostate specific antigen test for cancer screening purposes. Standard treatment options consist of surgery or definitive radiation therapy directed by clinical factors that are organized into risk stratification groups. Current clinical risk stratification systems are still insufficient to differentiate lethal from indolent disease. Similarly, a subset of men in poor risk groups need to be identified for more aggressive treatment and enrollment into clinical trials. Furthermore, these clinical tools are very limited in revealing information about the biologic pathways driving these different disease phenotypes and do not offer insights for novel treatments which are needed in men with poor-risk disease. We believe molecular biomarkers may serve to bridge these inadequacies of traditional clinical factors opening the door for personalized treatment approaches that would allow tailoring of treatment options to maximize therapeutic outcome. We review the current state of prognostic and predictive tissue-based molecular biomarkers which can be used to direct localized prostate cancer treatment decisions, specifically those implicated with definitive and salvage radiation therapy.

## INTRODUCTION

Prostate cancer is the most common cancer diagnosed in men in the United States. An estimated 217,000 new cases of prostate cancer were diagnosed in the United States in 2010 of which an approximate 85-90 percent were clinically localized at diagnosis [1]. Despite intense efforts to screen using clinical-pathologic factors, such as serum prostate specific antigen (PSA), and aggressively treat localized disease with surgery or radiation therapy, an unacceptable number of even early stage prostate cancer patients will ultimately succumb to their disease [2]. Prostate cancer as a whole still remains the second leading cause of cancer related death among men [1, 3]. Thus, key issues in the management of localized prostate cancer include not only the stratification of patients with aggressive disease from those with indolent cancer but also the development and improvement of therapies to treat localized prostate disease that is ultimately lethal. Taken a step further, a personalized approach would allow different treatment options to be tailored to specific traits of the patient and their prostate cancer to maximize clinical outcome. To address these issues, it is imperative to identify and validate new prognostic and predictive molecular biomarkers which can be used to direct localized prostate cancer treatment decisions. In this review, we catalog some of the work on tissue-based molecular biomarkers for prostate cancer, specifically those implicated with definitive and salvage radiation therapy treatment. We direct the reader to excellent reviews on prostate cancer biomarkers from other biologic specimens such as urine and blood [4, 5].

## CLINICAL FEATURES AND DEFINITIVE RADIATION TREATMENT FOR LOCALIZED PROSTATE CANCER

### Presentation

Clinically localized prostate cancer in the modern era usually presents initially as symptoms resulting in an abnormal finding on digital rectal examination (DRE) of the prostate and/or subsequent serum elevation of the biomarker PSA secondary to abnormal prostate exam or for screening purposes [6]. Serum PSA is currently the best molecular biomarker for prostate cancer when used for diagnostic and prognostic purposes [7]. Either of these clinical findings, abnormal DRE and elevated PSA, eventually prompt a prostate biopsy and can lead to the histological diagnosis of prostate cancer.

### Standard Clinical Staging Systems for Localized Prostate Cancer

Current standard of care paradigms use pretreatment patient and prostate cancer clinical factors for prognosis and to dictate treatment options [8]. Clinical factors that help risk stratify localized prostate cancer patients are the clinical cancer stage groupings which are determined by the anatomic Tumor-Node-Metastasis (TNM) staging system, the Gleason score or summed tumor grade and pretreatment serum PSA level [9-11]. Using this staging system, clinically localized prostate cancer patients can be assigned to four risk groupings: low-risk (Group I), intermediate-risk (Group IIA), high-risk (Group IIB) and locally advanced (Group III) [8, 9], each connoting progressively increasing chances of recurrence following definitive local therapy [12, 13]. In addition, patient clinical factors such as age and comorbid conditions are integrated with the risk groupings to determine the optimal therapy [8]. With the recent realization that within the low-risk group there is a sub-group of extraordinarily favorable patients as defined by the so-called “Epstein criteria” [14], the National Comprehensive Cancer Network (NCCN) have created a another localized prostate cancer risk group, termed very low-risk. Very low-risk patients are advised by the NCCN to undergo active surveillance and not pursue definitive local therapy if their life expectancy, disregarding their prostate cancer diagnosis, is less than twenty years [8].

### Radiation Therapy for Localized Prostate Cancer

Definitive treatment options for localized prostate cancer in general involve the use of surgery or radiation therapy [2, 8]. As mentioned above, active surveillance can also be a viable option for many localized prostate cancer patients in the very low-risk group [8, 14] and even for select patients of low [8, 15, 16] and intermediate-risk [8, 17]. Given the focus of this review is on radiation therapy biomarkers, we will only detail the use of radiation therapy further. Definitive radiation therapy typically involves the delivery of tumoricidal doses of high energy ionizing radiation to the whole prostate gland and sometimes to include the periprostatic tissues. Radiation is produced external to the body usually by a linear accelerator and directed *via *a number of different, but similar techniques, to pass through the patient with the highest dose deposited to the entire prostate [2, 8, 18]. Focal therapy of prostate cancer or treatments directed at less than the entire prostate is generally not advocated in the upfront setting unless as part of a clinical trial [16]. Another common form of radiation therapy used is the physical implantation of radioactive sources or brachytherapy, into the prostate which then decay and emit high energy radiation to the prostate gland [18]. The underlying mechanism for prostate cancer cell death from radiation therapy is radiation induced DNA damage. Radiation therapy damages prostate cancer DNA by rare direct ionization events or by the much more common indirect DNA damage from free radicals that occur as a by-product of the hydrolysis of water [19, 20].

High dose radiation therapy for localized prostate cancer can be very effective resulting in freedom from relapse of disease (or cure as determined by PSA) as high as 93% at 10 years in the most favorable or low-risk cases [21]. However, clinical experience with the use of radiation therapy alone in higher risk localized prostate cancer (intermediate [22, 23], high-risk [22, 24-27] and locally advanced groups [25-29]) results in relatively poor outcomes. This has lead to the standard approach of combination treatment with radiation therapy and hormone deprivation therapy (ADT) for variable duration, resulting in moderate improvements in clinical outcomes [30]. ADT is the surgical or pharmacologic depletion of testosterone to very low or castrate levels in prostate cancer patients [30]. The mechanism of action by which ADT increases radiation therapy effectiveness is complicated and not fully appreciated yet, but is thought to involve increased radiation-induced apoptosis, decreased prostate cell proliferation and perhaps suppression of systemic micrometastases [31-34]. Taken altogether, the standard radiation treatment recommendations for the localized prostate cancer risk groups that require definitive local therapy are as follows: (1) low-risk patients should receive definitive high dose radiation therapy alone; (2) intermediate-risk patients can receive high dose definitive radiation therapy alone, but more commonly a combination of definitive radiation therapy and short-term duration ADT are recommended; and (3) high-risk and locally advanced patients should receive a combination of definitive radiation therapy and long-term duration ADT [8]. It should be noted that intermediate-risk and high-risk patients are often treated with high-dose radiation therapy in combination with ADT because of the ability to safely deliver high dose radiation even though high level data in support of this approach is lacking. The target volumes treated with high-risk patients, sometimes referred to as *prostate alone* versus the inclusion of larger treatment volumes that include draining lymph nodes also known as *pelvic fields*, is controversial and will not be discussed further, but the reader is directed towards references on this subject [35-37].

## CLINICAL PREDICTIVE MODELS OR NOMOGRAMS FOR PROSTATE CANCER RADIATION THERAPY

To improve upon these more simplistic risk stratification tools in an effort to better guide treatment decisions, multiple groups have produced more elaborate predictive prostate cancer treatment models also sometimes referred to as *prostate cancer nomograms *[38-40]. These models incorporate the pretreatment patient and tumor clinical factors detailed above, but also can predict the effects of treatment related factors such as radiation therapy dose and the use of combined radiation therapy and ADT on clinical outcomes. Although, these prostate cancer nomograms can be very useful in the clinical setting, as is the case for any model, there are still questions regarding their general validity for all localized prostate cancer patients. An excellent review detailing the clinical experience, utility and shortcomings of these clinical predictive models for prostate cancer radiation therapy by Roach and colleagues is referenced [41].

## TISSUE BIOMARKERS FOR PROSTATE CANCER RADIATION THERAPY

While the clinical staging systems and predictive tools described above have helped tremendously to guide current standards in management of localized prostate cancer, they still do not uniformly distinguish indolent from lethal disease and are limited in revealing information about the genetic and biologic pathways driving these different disease phenotypes. In addition, they do not offer clear targets for the development of therapies to treat localized prostate cancer which are needed in patients with poor-risk disease. We believe molecular biomarkers may serve to bridge these inadequacies of traditional clinical factors.

A biomarker is as a factor, clinical or molecular, that can be correlated with a physiologic pathway, pathologic natural history or treatment response to a particular therapy [42]. Biomarkers can thus be very important tools to distinguish presymptomatic patients from unaffected healthy individuals such as with serum PSA screening, to monitor disease progression, to recognize those who are susceptible to adverse effects and to determine the efficacy of specific therapies. Biomarkers come in every imaginable flavor: clinical exam findings; physiologic parameters; results from imaging studies; and the focus of this review, molecules from biologic material such as body fluids or tissue specimens, are all biomarkers. The commonality between these diverse types of biomarkers is at the minimum they serve as surrogate measures for a pathophysiologic endpoint [43] and in the case of molecular biomarkers can also be a critical causative effector target for the desired process under scrutiny. Examples of the later, where predictive molecular biomarkers are also critical nodal targets of a tumor maintenance pathway are *c-KIT* mutations in gastrointestinal stromal tumors (GIST) [44, 45] and *Epidermal Growth Factor Receptor *(*EGFR*) mutations in non-small cell lung cancer (NSCLC) [46-49] that predict response to and are both targets of tyrosine kinase inhibitors (TKIs). For the remainder of the review we will restrict our use of tissue-based molecular biomarkers to surrogates of important prostate cancer clinical outcomes following radiation therapy.

### Prognostic versus Predictive Biomarkers

**Fig. (1):**
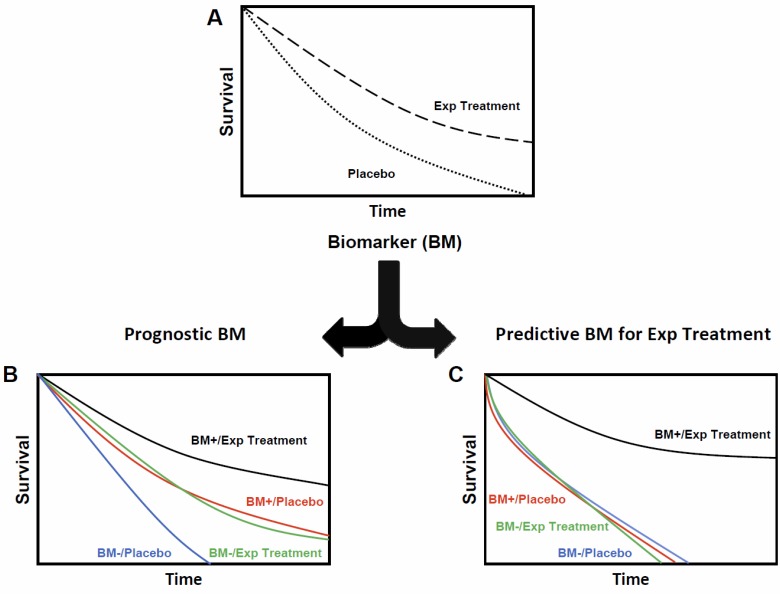
**Prognostic versus predictive biomarkers**. An idealized example of the interrogation of the prognostic versus predictive properties of a biomarker (BM) are shown. (**A**) An experimental treatment (Exp Treatment) is tested in a randomized controlled fashion against a placebo or control arm and shown to confer a survival advantage. A biomarker (BM) has been shown to correlate with improved benefit with the Exp Treatment in prior uncontrolled studies without a control arm. By segregating the groups based on their treatment arms and BM status (BM+/Placebo; BM+/Exp Treatment; BM-/Placebo; and BM-/Exp Treatment) it is possible to distinguish whether the BM is purely prognostic versus predictive. (**B**) The BM is purely prognostic and therefore independent of the treatment effect. The relative magnitude of the benefit from the Exp Treatment is similar for each BM group. (**C**) The BM is purely predictive for the Exp Treatment and all the benefit from the Exp Treatment is exhibited only for patients in the BM+ group.

Distinguishing biomarkers as prognostic versus predictive is not simply semantics, but denotes specific properties of each biomarker type for treatment response and clinical outcomes (Fig. **1**) [50]. A prognostic biomarker is correlated with an endpoint irrespective of therapy. In practice, prognostic biomarkers help guide treatment decisions made by clinicians. Using clinical risk based staging systems as an example, in general, low risk prostate cancer patients are treated with one modality, i.e. radiation therapy alone, while higher risk patients are directed towards combined modality treatments, i.e. radiation therapy and ADT [8]. However, purely prognostic biomarkers do not correlate with clinical outcomes to a specific treatment. In contrast, a predictive biomarker is correlated with an improvement or lack of improvement in clinical outcomes to a specific treatment. This prediction of outcome to a specific treatment is dependent on the status of the predictive biomarker. As cited above, predictive biomarkers have also been shown to be targets for therapy [44-49]. The determination of the prognostic versus predictive properties of a biomarker can be complex and is beyond the scope of this review (for an excellent reference on this topic [50]), however, one point is worth explicitly stating. The determination of the prognostic and predictive properties of a biomarker is best evaluated in a randomized clinical trial with a control group, because of the confounding effects present in most single-arm treatment trials (see Fig. **1**; this type of analysis is not possible without a placebo or control group). The need for a comparator or control group to determine predictive properties of a biomarker will restrict our review to the high-level prostate cancer radiation therapy trials performed by the Radiation Therapy Oncology Group (RTOG).

### Radiation Therapy Oncology Group (RTOG) Prostate Cancer Biomarker Studies

The RTOG is a long standing cooperative research groups which has conducted numerous prospective trials in the field of radiation oncology, including those pertaining to prostate cancer. Two seminal phase III randomized trials, RTOG 86-10 [25] and 92-02 [27], were conducted in high-risk localized and locally advanced prostate cancer patients to address the role of combination treatment of radiation therapy and ADT and duration of ADT (short-term versus long-term), respectively. These two trials have also been the source of biologic material, in the form of prostate specimens, which has allowed RTOG investigators to explore the prognostic and in some cases predictive power of a number of tissue-based molecular biomarkers (Table **1**). In order to understand the implications that these biomarkers may have on prognosis and predictive ability following radiation therapy with or without the addition of ADT, we will review the specifics of each trial below. Given the use of ADT in the vast majority of patients on these two trials, it should be noted that these tissue biomarkers are probably best described as surrogates of prostate cancer treatment with radiation and androgen deprivation therapy.

RTOG 86-10 was a randomized trial conducted in the pre-PSA era of radiation therapy (RT) alone versus radiation therapy and ADT for patients (n=471 randomized) with bulky tumors (≥25 cm2 by DRE) and T stage T2b–4. One of the original hypotheses for this trial was to determine whether ADT before and during radiation therapy may reduce tumor bulk and enhance tumor cell kill and thus improve tumor control and survival [51]. Thus, patients in the ADT arm were pharmacologically castrated using goserelin (luteinizing hormone-releasing hormone agonist) and flutamide (anti-androgen) for 2 months before and 2 months during radiation therapy. The most updated results of this trial [25], in keeping with an earlier report [51], demonstrated statistically significant improvements in 10-year disease-specific mortality or death from prostate cancer (23% *vs.* 36%; *p* = .01), distant metastasis (35% *vs.* 47%; *p* = .006), disease-free survival (11% *vs.* 3%; *p* < .0001), and PSA failure (65% *vs.* 80%; *p* < .0001), all favoring the combination arm.

RTOG 92-02 is the largest randomized trial of prostate cancer that tested the optimal duration of ADT when combined with radiation therapy for prostate cancer patients (n=1554 randomized) with T2c–4 primary tumors and PSA <150 ng/ml. The control arm was based on RTOG 86-10, ADT using goserelin and flutamide for 2 months before and 2 months (or short-term ADT = STAD) during radiation therapy (RT + STAD). The experimental arm added 24 months of additional goserelin alone also known as long-term ADT (LTAD). The mature results (median 11 years of follow-up) [27] from this trial demonstrate the superiority of RT + LTAD over RT + STAD for patients with high-risk/locally advanced prostate cancer for 10-year disease-specific survival (83.9% *vs.* 88.7%; *p* = .0042), local progression (22.2% *vs.* 12.3%; *p* < .0001), distant metastasis (22.8% *vs.* 14.8%; *p* < .0001), disease-free survival (13.2% *vs.* 22.5%; *p* < .0001) and PSA failure (68.1% *vs.* 51.9%; *p* ≤ .0001). Neither RTOG trial demonstrated an overall survival benefit. Multiple reasons have been postulated to explain this lack of overall survival benefit for RTOG 92-02 including the advanced age of the patients and the large enrollment of lower Gleason score (GS ≤7) patients. Regardless, hypothesis generating subset analysis has demonstrated an overall survival benefit for patients with GS 8-10 (31.9% *vs.* 45.1%; *p* = .0061). These RTOG trials helped form the rationale for the use of combination radiation therapy and LTAD for high-risk and locally advanced prostate cancer patients. In addition, they have been an incredible resource to study candidate prognostic and predictive biomarkers for prostate cancer radiation therapy and androgen deprivation therapy.

### p53 – Prognostic and Predictive

The p53 protein encoded by the *TP53* gene is the most commonly mutated gene in human cancer. The p53 tumor suppressor protein has multifaceted roles as a stress-responsive transcription factor to a variety of intracellular and extracellular insults, notably including those that are produced by radiation therapy [52]. Preclinical studies in prostate cancer cells suggest that p53 loss of function results in radiation resistance [53, 54]. As a biomarker, p53 accumulation or *TP53* mutation has been associated with poor prognosis in multiple different cancer types. Other groups have also looked at the role of p53 as a biomarker for prostate cancer patients treated with radiation therapy, often as part of a heterogeneously treated group, producing as expected varied results [55-61]. Many of these groups did not have the benefit of tissue collection from a prospective randomized clinical trial such as with the RTOG with a control group of patients, thus as explained above negating the ability to look at predictive power.

Give these circumstances, p53 was an ideal biomarker candidate for the RTOG to test for prognostic and predictive power using the biospecimens from RTOG 86-10 [62] and 92-02 [63]. From RTOG 86-10, abnormal accumulation of p53, defined as ≥20% positive nuclear staining using immunohistochemistry (IHC), was present in 23/129 (18%) patient tumor samples available from the original 471 enrolled on the trial (or only 27%). The p53 biomarker was independently prognostic for increased distant metastasis (RR = 2.15, *p* = 0.04), poor progression-free (RR = 2.45, *p* = 0.003) and overall survival (RR = 2.34, *p* = 0.02) for the entire group (RT and RT + STAD arms). There was also a curious finding that the p53 biomarker seemed to predict increased metastasis in the RT+STAD suggesting that patients with abnormal p53 biomarker status do not react favorably to RT + STAD. This is likely an artifact from confounding due to the small numbers of cases with abnormal p53 biomarker status (12 with RT and 11 with RT + STAD) from a subset (only 27% of the original trial enrollment) that was not representative of the original trial cohort. Nonetheless, this was arguably the first study to demonstrate a clear prognostic role for the p53 as a molecular biomarker for prostate cancer patients treated with radiation therapy.

For RTOG 92-02 [63], tissue was available from 379 STAD + RT cases and 398 LTAD + RT cases for a total of 777 cases. Abnormal p53 was present in 168/777 (22%) patient samples and was an independent prognostic factor correlated with increased distant metastasis (HR = 1.72, *p* = 0.013) and cause-specific mortality (HR = 1.89, *p* = 0.014). When checking for the predictive power of p53 expression between RT + STAD and RT + LTAD treatment groups, the RTOG demonstrated that for patients treated with RT + STAD, p53 was a predictive biomarker associated with increased cause-specific mortality (HR = 3.81, *p* = 0.009). This suggest that p53 is a both a prognostic and predictive biomarker for high-risk/locally advanced prostate patients receiving radiation therapy and those with abnormal p53 status should be treated with RT + LTAD. Some caveats regarding these p53 biomarker studies and IHC data driven biomarker studies in general need to be introduced before proceeding further. The methods by which samples are scored: (1) manual methods versus quantitative image analysis; and (2) choice of cut-points, can dramatically influence the prognostic and predictive associations determined. Using the p53 biomarker as an example, a second analysis using RTOG 92-02 specimens and using quantitative image analysis systems with different cut-points was performed for the p53 biomarker [64]. The results of this second analysis resulted in weaker prognostic association with overall survival (RR ~ 1.3 *p* =0.02) and was no longer prognostic for distant metastasis or prostate cancer death. Hence great detail should be paid to the methods and even greater caution should be emphasized in the interpretation of biomarker data. Ultimately, these data are hypothesis generating at best and need themselves to be prospectively answered in a clinical trial. After acknowledging these caveats, these RTOG data in two different but complimentary patient data sets, reanalyzed twice in the case of RTOG 92-02, suggest that p53 appears to be a prognostic molecular biomarker. Furthermore, abnormal p53 biomarker status appears to predict patients that may benefit from RT + LTAD.

### DNA Ploidy - Prognostic and Predictive

DNA ploidy or chromosome complement is a crude measure of genomic instability, a hallmark of tumorigenesis [65], and in most cases has been correlated as a biomarker portending worse prognosis for prostate cancer [66-79]. An even more limited number of studies have produced mixed results after examining DNA ploidy as a biomarker for prostate cancer patients treated with radiation therapy [80-85].

The RTOG using 149 specimens from trial 86-10 examined DNA ploidy and found 55/149 (37%) patients to be non-diploid [86]. This non-diploid biomarker status was independently prognostic for worse 5-year overall survival (70% versus 42%, *p* = 0.031), but not any other clinical endpoints. The reduced overall survival in the absence of an increase in other endpoints was explained by what appeared to be increased resistance to salvage ADT by patients with non-diploid tumors who had been treated with RT+STAD. The DNA ploidy biomarker showed a predictive interaction with the patients on the RT + STAD arm of RTOG 86-10 demonstrating a worse overall survival (*p* = 0.02) and worse survival following salvage ADT with non-diploid biomarker status (*p* = 0.01), which was not present in the control RT alone arm (*p* = 0.56 and *p* = 0.37, respectively). These results were have not been duplicated in published form using the RTOG 92-02 dataset.

### COX-2 – Prognostic and Predictive

Inflammation as a driving event in prostate tumorigenesis and tumor progression is acknowledged [87]. Mediators and modifiers of inflammation such as cyclooxygenase-2 (COX-2) have been studied with great interest. Potential roles of COX-2 in tumor related processes such as tumorigenesis, angiogenesis [88-90] and radiation treatment resistance makes this an attractive biomarker candidate and potential therapeutic target [91-95].

From a cohort of 1554 patients from RTOG 92-02, 586 patient samples (270 STAD + RT cases and 316 LTAD + RT) were analyzed by COX-2 IHC [96]. By multivariate analysis COX-2 staining was an independent prognostic factor of distant metastases (*p *< 0·02) as a continuous factor or by using a cut-point. COX-2 was also prognostic for PSA failure [using two separate definitions (ASTRO: HR = 1·073, *p*=0·008; Phoenix: HR = 1·073, *p*=0·014) when COX-2 staining was considered as a continuous variable. Interestingly, COX-2 overexpression was associated with worse PSA failure (by ASTRO definition) for those on the control arm of RT + STAD but not those on the RT + LTAD (*p* = 0.002), suggesting that COX-2 may serve as a predictive biomarker for length of ADT therapy in high-risk and locally advanced prostate cancer patients.

### Protein Kinase A – Prognostic and Predictive

The protein kinase A (PKA) is a holoenzyme of two regulatory and two catalytic subunits dependent on cyclic AMP [97] that is overexpressed in a variety of cancer types and associated with poor prognosis [98-100]. Knockdown of the gene encoding PKA enhanced the response of androgen-sensitive prostate cancer cells to ADT with or without RT and androgen-insensitive cells to RT [101].

PKA overexpression was determined for 80/456 (17.5%) samples from RTOG 86-10 [102]: 36 men from RT alone control arm and 44 from the RT + STAD arm. The PKA IHC was analyzed using both manual and image analysis methods. PKA expression was found to be very weakly prognostic for PSA failure (using image analysis as a continuous variable, HR = 1.01, *p* = 0.03), but stronger for local recurrence (using image analysis with a cut-point of >2 using a scale of 0-3, HR = 3.66, *p* = 0.002), and distant metastases (manual analysis with a cut-point of >2 using a scale of 0-3, HR = 2.27, *p* = 0.018). No interaction between PKA biomarker expression and the treatment arms of RTOG 86-10 was found.

Examining the RTOG 92-02 biorepository for PKA overexpression resulted in IHC analysis on 161 patients from the STAD + RT control arm and 152 patient samples from the LTAD + RT arm (313 total cases) [103]. In contrast to RTOG 86-10, the IHC was analyzed using both manual and image analysis methods. PKA overexpression by IHC was independently prognostic for PSA failure (*p* ≤ 0.01), local recurrence (*p* < 0.05), and distant metastases (*p* < 0.01) confirming the results from RTOG 86-10. In contrast to RTOG 96-10, when the investigators examined for potential interactions between PKA biomarker expression and the treatment arms of RTOG 92-02 they found significant prediction of the outcome for patients treated on the RT + LTAD arm. Low PKA expression was predictive of decreased PSA failure (HR = 0.54, *p* = 0.0003), local recurrence (HR = 0.31, *p* = 0.007), distant metastases (HR = 0.23, *p* = 0.003) and prostate specific death (HR = 0.25, *p* = 0.005). These results suggest that the benefits from RT + LTAD are the most pronounced in groups of patients with low PKA expression and consequently, novel strategies may be needed for patients whose tumors have high PKA levels.

### BCL-2/BAX – Prognostic and Predictive

The BCL-2 family are dimeric proteins that are defined by containing Bcl-2 homology (BH) domains and act as anti- or pro-apoptotic regulators [104, 105]. BAX is a pro-apoptotic cytosolic family member that following initiation of apoptotic signaling inserts itself into the outer mitochondrial membrane contributing to the release of cytochrome c and other pro-apoptotic factors. In contrast, BCL-2 is the prototypical anti-apoptotic protein that exerts its effects by titrating out pro-apoptotic proteins such as BAX. The relative amounts of BCL-2 and/or BAX has been shown to correlate with tumor aggressiveness and radiation resistance in prostate cancer [58, 106-112].

The RTOG examined the relative levels of BCL-2 and BAX by IHC in both 86-10 [113] and 92-02 [114] trials and correlated with prognostic and predictive ability for clinical outcomes. The findings from RTOG 86-10 were negative and we will instead focus on those from RTOG 92-02. BCL-2 was positive in 45.6% (229/502) of cases and BAX expression altered in 53.9% (185/343) of cases. BCL-2 was not found to correlate to any of the clinical end points. However, altered BAX expression was independently prognostic for any type of disease failure (RR = 1.43, *p* = 0.0226). Next the investigators analyzed the BCL-2 and BAX staining as composite groups given their biologic interaction for apoptosis regulation. When examining the composite group of positive BCL-2 and/or altered BAX, this biomarker group was independently prognostic for any failure (RR = 1.45; *p* = 0.046) and PSA failure (ASTRO definition, RR = 1.60; *p* = 0.036). Lastly, this same biomarker group was predictive for worse 5-year PSA failure when comparing RT + STAD versus RT + LTAD (61% versus 24%, *p* < 0.0001).

### p16 – Prognostic

The *CDKN2A* tumor suppressor gene is commonly homozygously deleted in a wide variety of primary tumors cancer and cell lines which helped lead to its positional cloning [115, 116]. *CDKN2A *encodes for p16 which is a cyclin-dependent kinase inhibitor (CDKI) and a critical component of the pRB/p16 tumor suppressor axis. This axis forms a critical regulatory system for the G1-S-phase transition and is found to be dysregulated in a majority of human cancers [117].

A subset of tissue blocks (67/471) from RTOG 86-10 were examined by IHC for decreased p16 and pRB levels [118]. Using cut-off values of ≤25% for p16 and ≤20% for pRB staining cells, RTOG investigators found that 18/67 (27%) and 54/67 (81%) samples demonstrated decreased p16 and pRB levels, respectively. Decreased p16 expression was prognostic for higher risk of local progression (*p* = 0.0035), distant metastasis (*p* = 0.026) and prostate cancer death (*p* = 0.01). Decreased pRB levels were oddly prognostic for decreased risk of death from prostate cancer (*p* = 0.03). The predictive power of the p16 and pRB biomarkers were not tested for interaction with the treatment arms of RTOG 86-10 likely because of the small subset sample sizes.

A follow-up study using samples from RTOG 92-02 was performed examining the p16 biomarker only and by utilizing a quantitative image analysis system [119]. Tissue was available from 285 STAD + RT and 327 LTAD + RT for 612 total cases. The p16 biomarker was prognostic for distant metastases (*p* = 0.0332) similar to RTOG 86-10. Tests for interaction between p16 biomarker levels and treatment effect revealed no significant interactions. However, this study represented a sub-group that may not have represented the original cohort of patients of RTOG 92-02 and investigators used arbitrary cut-points with their quantitative analysis perhaps limiting the power to detect predictive interactions with p16 and the treatment arms. In summary, the data from RTOG 86-10 and 92-02 suggest that p16 is a prognostic marker for distant metastasis and perhaps local progression and prostate cancer death. Confirmatory studies are warranted before p16 can be used as prognostic biomarker for prostate cancer patients and no data is available to suggest that p16 is predictive for who should receive RT alone, RT + STAD or RT + LTAD.

### Ki-67 – Prognostic 

The Ki-67 antigen is a biomarker used to determine proliferative rate for many other types of tumors [120], as well as been shown to correlate with clinical outcomes of prostate cancer patients treated with surgery or radiation therapy [80, 108, 109, 121-128]. One previous study had suggested a Ki-67 index of ≥3.5% was prognostic for PSA failure following radiation therapy [126].

RTOG 86-10 samples were used to validate this cut-point for Ki-67 staining [129]. Diagnostic samples from 108 patients were available for Ki-67 analysis, 60 cases from the RT alone arm and 48 patient samples from RT + STAD arm with a median follow-up of 9 years. Using the 3.5% cut-point Ki-67 was independently prognostic for 5-year risk of distant metastases (13.5% versus 50.8%; *p* = 0.0005) and prostate specific survival (97.3% versus 67.7%; *p* = 0.0039). In a separate analysis from the same study, the investigators also found the 7.1% cut-point to be prognostic on univariate testing for distant metastases and prostate specific survival. No tests for Ki-67 interaction with the treatment arms of RTOG 86-10 were performed to allow predictive assessment.

Ki-67 staining was studied in the RTOG 92-02 data set using 537 patient specimens, 257 from the RT + STAD arm and 280 from RT + LTAD arm [130]. The investigators looked at the Ki-67 staining as both a continuous variable and also using their 3.5% and 7.1% cut-points from above. As a continuous variable Ki-67 staining was independently prognostic for PSA failure (*p* = 0.0445), distant metastases (*p* < 0.0001), prostate specific survival (*p* < 0.0001) and overall survival (*p* = 0.0094). The 7.1% cut-point was also independently prognostic for distant metastases (*p* = 0.0008) and prostate specific survival (*p* = 0.017). No definitive predictive correlations were made with Ki-67 staining and the treatment arms of RTOG 92-02. The investigators did identify some possible groups of prostate cancer patients that may not require LTAD by looking at various permutations of the 7.1% Ki-67 cut-point with other traditional clinical risk factors. In a separate study this same IHC staining was duplicated, but the RTOG investigators used quantitative image analysis and different cut-points [64]. This duplicate analysis found Ki-67 to again be independently prognostic for distant metastases (*p* < 0.0001), prostate specific survival (*p* = 0.0007) and overall survival (*p* = 0.01). Taken altogether, the Ki-67 biomarker was found to be strongly prognostic in two separate validation sets for distant metastases and prostate specific survival.

### MDM2 – Prognostic

The human orthologue of the murine double minute (MDM2) oncoprotein is a negative regulator of p53 that regulates p53 degradation and represses p53 transcriptional targets [131, 132]. MDM2 also appears to confer androgen independent survival and radioresistance to prostate cancer cells *via *p53-dependent and independent pathways [133-135]. As described above, p53 as a biomarker has been shown to have promising prognostic and predictive potential for patients treated on RTOG 86-10 and 92-02. Only a limited number of studies had examined MDM2 as a biomarker in prostate cancer specimens with mostly uncontrolled cohorts [136, 137].

When RTOG investigators looked at MDM2 by IHC as a biomarker in the 86-10 cohort, they were able to examine 108 patients (62 from the RT arm and 46 from the RT + STAD arm) and found at least 47 specimens (44%) that had MDM2 overexpression. The investigators analyzed their data using a cut-point of >5% nuclear staining by manual analysis and with at 3% cut-point using a quantitative method, but did not find that MDM2 was independently prognostic or predictive for any clinical outcome [138].

A larger study using RTOG 92-02 specimens (589 total) of MDM2 IHC and quantitative image analysis was performed (64). MDM2 was found to be independently prognostic for distant metastases (*p* = 0.02) and overall survival (*p* = 0.003). In addition, the group performed a concurrent analysis of the individual prognostic power of the proliferative index by Ki-67 IHC and p53 overexpression which we described above separately. Combining MDM2 overexpression and high Ki-67 resulted in increased prognostic significance for distant metastasis (*p* < 0.0001), prostate cancer death (*p* < 0.0001) and overall survival (*p* = 0.0002). However, no predictive correlations could be made between MDM2 and Ki-67 and any of the clinical outcomes depending on treatment arm. MDM2 individually and particularly in combination with Ki-67 appears to be a robust molecular biomarker for prostate cancer patients treated with radiation therapy.

### Survivin – Prognostic

Survivin is a member of the inhibitor of apoptosis (IAP) family and inhibits caspase activation preventing apoptosis. Survivin is almost exclusively expressed during embryogenesis [139], but is found to be overexpressed in a wide variety of tumors types including prostate cancer, where it is associated with prognosis [140, 151]. Survivin also mediates paclitaxel and androgen deprivation resistance in prostate cancer cells [140, 152].

The RTOG examined nuclear survivin as a biomarker using the 86-10 cohort from 68 patient samples [153]. The samples analyzed were not representative for prostate cancer survival and distant metastases compared to the original 86-10 larger cohort of 456 patients and thus the results below may not generalize. With these caveats, nuclear survivin was independently prognostic for improved prostate cancer survival (HR = 0.36, *p* = 0.0173) and overall survival (HR = 0.46, *p* = 0.0156). Survivin exists in cytoplasmic and nuclear pools [154], with cytoplasmic survivin associated with poor outcomes [150]. Therefore, another analysis scored for cytoplasmic staining on 65 patient samples from trial 86-10. An association for increased local recurrence and cytoplasmic surviving was established, but was not upheld on multivariate analysis. Further analyses of survivin as a biomarker are warranted with a larger data such as with RTOG 92-02.

### Other Biomarkers Tested on RTOG 86-10 and 92-02 Trials

Five additional molecular biomarkers where tested using the patient samples from RTOG 86-10 or 92-02. The nuclear factor-κB [155], chemokine receptor CXCR4 [155], STAT3 [156] and CAG triplet repeat number located within the gene for the androgen receptor (AR) [157] were examined using materials from ROG 86-10. A very small subset of patients from RTOG 92-02 were examined for racial polymorphisms of the cytochrome P450 3A4 (*CYP3A4*) gene responsible for androgen metabolism [158]. The hypotheses for studying these molecules were all well conceived and experiments performed in keeping with the high standards of the studies as detailed above. However, these studies were either under-powered, demonstrated no/weak or paradoxical associations with clinical outcomes and so will not be discussed. The reader is directed to these original publications for further descriptions of these works.

## SELECT NON-RTOG PROSTATE CANCER TISSUE BIOMARKER STUDIES

Two additional non-RTOG tissue biomarker studies using patient samples from randomized dose escalation studies conducted by the Medical Research Council (MRC) from the United Kingdom will also be reviewed [159, 160]. The MRC RT01 trial (n=843) and the phase II pilot predecessor trial (n=127) were almost identical in design. Patients received 3–6 months of ADT followed by prostate radiation therapy that was randomly assigned to 64 Gy versus 74 Gy. In their initial study, the MRC investigators examined hypoxia inducible factor-1 alpha (HIF-1 alpha), vascular endothelial growth factor (VEGF) and osteopontin. These three markers have been implicated in intratumoral hypoxia which has been shown to have a role in modulating radiation response in multiple tumor types [161]. HIF-1 alpha is a transcription factor that is stabilized under hypoxic conditions and induces a number of genes, such as *VEGF* resulting in tumor progression, angiogenesis, and metabolic changes, making it an ideal biomarker and potential therapeutic target [162]. The MRC were able to obtain 201 patient samples from the randomized studies (n=103 from the 64 Gy arm and n=98 from the 74 Gy arm) and perform IHC from tissue microarrays. The predictive properties of these hypoxia tissue biomarkers were not analyzed. However, HIF-1 alpha (HR = 1.46, *p* = 0.02) and VEGF (HR = 1.45, *p* = 0.008) were both found to be independently prognostic for PSA failure (nadir + 2 ng/mL).

The MRC investigators also examined some of the same tissue biomarkers as the RTOG: Bcl-2, p53 and MDM2, and where able to replicate that Bcl-2 was both a prognostic (HR = 3.57, *p* = 0.001) and interestingly, a predictive biomarker for radiation therapy with ADT. Using the same tissues as above, they specifically showed that patients with Bcl-2 positive tumors demonstrated improved 7-year biochemical control (41% versus 61%, *p *= 0.0122) with escalated doses of radiation therapy (64 Gy versus 74 Gy) and ADT. Whereas patients with Bcl-2 negative tumors did not demonstrate any improvement with dose escalated radiation therapy (*p* = 0.423), suggesting that Bcl-2 status could be used to predict patients requiring more aggressive treatment in the form of radiation dose escalation or longer duration of ADT.

## MOLECULAR BIOMARKERS FOR ADJUVANT AND SALVAGE RADIATION THERAPY

As mentioned above, localized prostate cancer can be treated successfully with definitive local therapies such as surgery or radiation therapy. Surgery in the form of a radical prostatectomy is the gold standard by which other local therapies are measured [2]. Unfortunately, in the United States approximately 30,000 men per year will still experience PSA failure following their surgery with many of these men ultimately developing metastases [163, 164]. Radiation therapy to the prostatic fossa or surgical bed is the only potentially curable remaining option for these men. Radiation therapy to the prostatic fossa shortly after surgery in the absence of known disease, i.e. undetectable PSA, based on known poor pathologic prognostic factors such as extra-prostatic disease and positive surgical margins is known as adjuvant radiation therapy (ART). Three phase III randomized trials have demonstrated improvement in clinical outcome for patients undergoing ART who display these poor pathologic risk factors [165-167] (and we refer readers to an excellent review of these seminal studies [168]). However, the number of men needed to treat for a survival benefit is still too high (12 men treated to save 1 life) [165]. No published studies on these phase III randomized trials or any single-institution cohort experiences are available examining molecular biomarkers for ART. The phase III trials [165-167] in particular provide an optimal opportunity to assess the prognostic and more importantly the predictive power of molecular biomarkers for ART. We expect these types of molecular biomarker studies to provide important tools to personalize ART for patients in the future.

The use of radiation therapy following surgery for clinically persistent or recurrent disease, typically a detectable PSA, is known as salvage radiation therapy (SRT). The success of SRT is highly variable [169, 170]. Similar to initial diagnosis, additional clinical tests including imaging to distinguish the extent of disease are sometimes warranted [8, 168, 171]. However, in general these additional clinical tests are not helpful at determining whether men with persistent prostate cancer will benefit from SRT. Clinical and pathologic factors have been identified to help refine the group of men likely to benefit [169, 172], but these risk stratification groups are still crude. Similar to definitive prostate cancer treatment, SRT prostate cancer nomograms have been created that also incorporate treatment related factors such as SRT dose and the use of ADT [170]. Again, the use of molecular biomarkers for the salvage setting should similarly improve treatment decisions and identify potential targets for therapy. However, prognostic molecular biomarkers for SRT, although further along than for ART, are still in their infancy and consist of only a few single-institution, retrospectively gathered cohort studies. A subtle but important implication of ART-SRT biomarkers; they may be surrogates for presence or absence of metastatic disease at the time of ART-SRT and/or they may be surrogates for the intrinsic response of disease to radiation therapy. Lastly, since these are all single-arm biomarker studies, no appreciation of predictive power can be made. We will review these few studies in detail below.

### p21

The *CDKN1A* gene encodes the p21 cyclin-dependent kinase inhibitor 1 (CDKI1) protein. The p21 protein is a critical regulator of cell cycle progression through G1 and forms a potent G1 checkpoint with the p53 tumor suppressor following various cellular stressors including radiation. Previous studies on prostatectomy specimens have indicated that p21 overexpression correlated with worse prognosis possibly mediated by the ability of p21 to inhibit genotoxic cell death from apoptosis. Riguad *et al.* [173] examined p21 as a biomarker for prognosis in 74 patients with a median age of 65 years (range 48-74) who received an average of 60 Gy SRT (range 50–70) 34.5 months (mean 42, range 4–112) following their surgery. Most had a pGleason score ≤ 7 (63/74), extraprostatic disease ≥pT3 (50/74) and positive surgical margins (48/74). The median pre-SRT PSA was 0.8 ng/ml (range 0.3–36.8). The p21 levels were assessed by IHC and scored manually demonstrating that 15/74 (20%) of the specimens overexpressed p21. The p21 biomarker was not correlated in this group with PSA failure following surgery before SRT. However, p21 was found to be independently prognostic for PSA failure (defined as > 0.3 ng/ml) following SRT when controlling for other variables such as pre-SRT PSA and pGleason score (*p* = 0.004). Risk stratification using p21 negative status and pre-SRT <1 ng/ml levels resulted in a subset of patients with a 5-yr PSA failure free survival of 83%. This group also conducted a concurrent IHC biomarker analysis of p53 and another CDKI, p27, but found no prognostic associations with their cohort of SRT patients.

### E-Cadherin

E-cadherin is associated with tumor invasiveness, metastatic dissemination, and poor patient prognosis [174, 175] including in prostate cancer [176-181]. E-Cadherin is a single-span transmembrane glycoprotein that establishes homophilic interactions with adjacent E-cadherin molecules expressed by neighboring cells assisting in formation of adherens junctions [182, 183]. Preclinical data suggests that knock-down of E-cadherin is sufficient to confer a phenotype of epithelial-mesenchymal transition (EMT) to normal and malignant cells and these cells that have undergone EMT are subsequently chemoresistant [184]. Some preclinical and clinical data suggest an EMT phenotype may confer a worse prognosis for prostate cancer patients [185]. Ray *et al.* [186] examined a small cohort of 37 patient samples for abnormal localization of E-cadherin by IHC who had been treated with a median of 68.4 Gy (range 64.8-70.2) SRT. Clinical characteristics of this cohort were most men had a pGleason score ≤ 7 (29/37), extraprostatic disease ≥pT3 (25/37) and positive surgical margins (24/37). Most patients had pre-SRT PSA that was <1 ng/ml (29/37). They found 68% (25/37) of their cohort had abnormal localization of E-cadherin following manual analysis of their IHC. Aberrant E-cadherin was an independent prognostic biomarker in this SRT population and conferred a worse PSA failure rate [HR = 3.8, *p* = 0.03; (defined as > 0.2 ng/ml greater than the nadir)]. 

### Ki-67

Ki-67 staining index as a marker of proliferation and use as a biomarker has demonstrated impressive independent prognostic ability in definitive cases of prostate cancer as described above and is a logical molecular biomarker candidate in the salvage setting. A group from the Mayo Clinic [187] assayed their a cohort of 147 patients treated with SRT (average 66.6 Gy) using Ki-67 IHC with techniques similar to those used above in the RTOG 92-02 trial. Other germane clinical characteristics of this cohort were most men had a pGleason score ≤ 7 (115/147), extraprostatic disease ≥pT3 (97/147), positive surgical margins (87/147) and follow-up was 6.2 years on average (range 0.6-14.7 years). The average pre-SRT PSA was 0.05 ng/ml (range 0.1-15.3). The average Ki-67 staining index was 2.5 in their cohort (range 0-20.9). High Ki-67 (as defined as >4) was an independent prognostic biomarker in this SRT population and conferred a worse PSA failure rate [RR = 2.02, *p* = 0.005; (defined as > 0.4 ng/ml or greater than the nadir)]. These SRT data and the RTOG studies in combination suggest that Ki-67 staining may be a general prognostic biomarker for prostate cancer.

### B7-H3 

The B7 ligand (encode by *CD276*) is a member of the B7 family of immuno-moldulation molecules [188-191]. B7-H3 functions are likely context dependent and can involve inhibition or stimulation of the immune response [192-195], but overexpression of B7-H3 has been correlated to worse clinical outcomes in prostate cancer [196]. The same group of investigators from the Mayo Clinic analyzed their SRT cohort of patients above for B7-H3 overexpression using IHC [197]. Manual scoring was performed and a majority of the tumors expressed B7-H3, but the intensity varied with 49 (33%) patient tumors showing weak B7-H3 staining, 70 (47%) with moderate B7-H3 intensity and 29 (20%) with strong B7-H3 staining. Interestingly, strong B7-H3 staining was independently prognostic for more likely PSA failure following SRT (RR = 2.87, *p* = 0.003).

## FUTURE PERSPECTIVE

The immediate future for the existing tissue-based molecular biomarkers as we have detailed above, is their prospective validation in clinical trials. The creation of integrative clinical-biomarker nomograms is on the near horizon for prostate cancer radiation therapy [41]. Combining the prognostic and predictive abilities of molecular biomarkers with other clinical factors that are independently prognostic and/or predictive is the next straightforward extension of current nomograms. We have focused our review on tissue-based molecular biomarkers, but an explosion of data is emerging from biomarker studies in other specimen types [4, 5]. In addition, imaging**-**based biomarker research is provocative and has the potential to further augment the tools for directing prostate cancer therapy [198]. Ultimately, the future of prostate cancer biomarkers, and medical biomarkers in general, will be a multiscale [199] and personalized approach that integrates genomic [200], transcriptomic [201], proteomic [202] and metabolomic [203] data to provide direction for personalized prostate cancer management.

## Figures and Tables

**Table 1.** Selected Prostate Cancer Radiation Therapy Biomarker Studies

**DEFINITIVE RADIATION THERAPY**	**BIOMARKER**	**PROGNOSTIC**	**PREDICTIVE**	**REFERENCE**
	p53 DNA	DM, PFS^1^, CSM^2^ & OS^1^	CSM^2^	[62-64]
	DNA Ploidy	OS^1^	OS^1^	[86]
	COX-2	BF^2^, DM^2^	BF^2^	[96]
	Protein Kinase A	BF, LF & DM	BF^2^, LF^2^, CSM^2^ & DM^2^	[102, 103]
	BCL-2/BAX	PFS^2^ & BF	BF	[113, 114, 159]
	p16	LF^1^, DM & CSM^1^		[118, 119]
	Ki-67	BF^2^, DM, CSM & OS^2^		[64, 129, 130]
	VEGF/HIF-1 alpha	BF^3^		[160]
	MDM2	DM^2^		[64, 138]
	Survivin	CSM^1^ & OS^1^		[153]
**SALVAGE RADIATION THERAPY**	p21	BF		[173]
	Ki-67	BF		[187]
	E-cadherin	BF		[186]
	B7-H3	BF		[197]

1 Found only in RTOG 86-10

2 Found only in RTOG 92-02

3 Found only in MRC RT01

BF – biochemical or PSA failure; LF – local failure; DM – distant metastases; PFS – progression free survival; CSM – cause specific mortality; OS – overall survival.
